# The Outcome of Progressive Uterine Sarcoma with Potential Bone Involvement

**DOI:** 10.1055/s-0042-1757285

**Published:** 2022-10-28

**Authors:** Akram Al-Ibraheem, Ahmed S. Abdlkadir

**Affiliations:** 1 Department of Nuclear Medicine, King Hussein Cancer Center, Amman, Jordan

**Keywords:** endometrial stromal sarcoma, uterine sarcoma, bone metastasis, nuclear medicine

## Abstract

High-grade endometrial stromal sarcoma (HGESS) is a very rare kind of uterine tumor that accounts for less than 2% of all uterine cancers. The usual sites of metastasis are the abdomen and lung, while bone metastasis is very rare, with only a few cases reported till now. We present a case of HGESS with bone metastasis and review the literature.

## Introduction

Endometrial stromal sarcoma (ESS) is a rare uterine malignancy that arises from endometrial stromal cells. It accounts for less than 2% of all uterine malignancies. 1 It is, nevertheless, the second most frequent kind of uterine interstitial tumor. 1 2

Based on clinical, pathological features, and molecular genetic studies of ESS, the World Health Organization classified ESS into three types as follows: (1) low-grade ESS (LGESS), (2) high-grade ESS (HGESS), and (3) undifferentiated uterine sarcoma (UUS). 3 The limited clinical data on the symptoms of HGESS and the lack of biomarkers contribute to the delayed diagnosis. Moreover, the overlapping symptoms of uterine fibroids may often lead to misdiagnosis.

## Case Report

In January 2021, a 58-year-old female patient presented to the gynecologic department with postmenopausal vaginal bleeding for 9 days. Pelvic magnetic resonance imaging (MRI) showed multiple uterine fibroids with cystic degeneration and heterogeneous enhancement distorting the uterine cavity. She was misdiagnosed and treated as a case of uterine fibroids.

Symptoms began to progress over time, and on further gynecologic consultation, it was decided that the patient should have surgical intervention. She underwent a total abdominal hysterectomy with bilateral salpingooophorectomy in April 2021. Pathology showed HGESS, limited to the uterine wall with no involvement of the parametrium or cervix. The tumor was pathologically staged as pT1aNx.

**Fig. 1:**
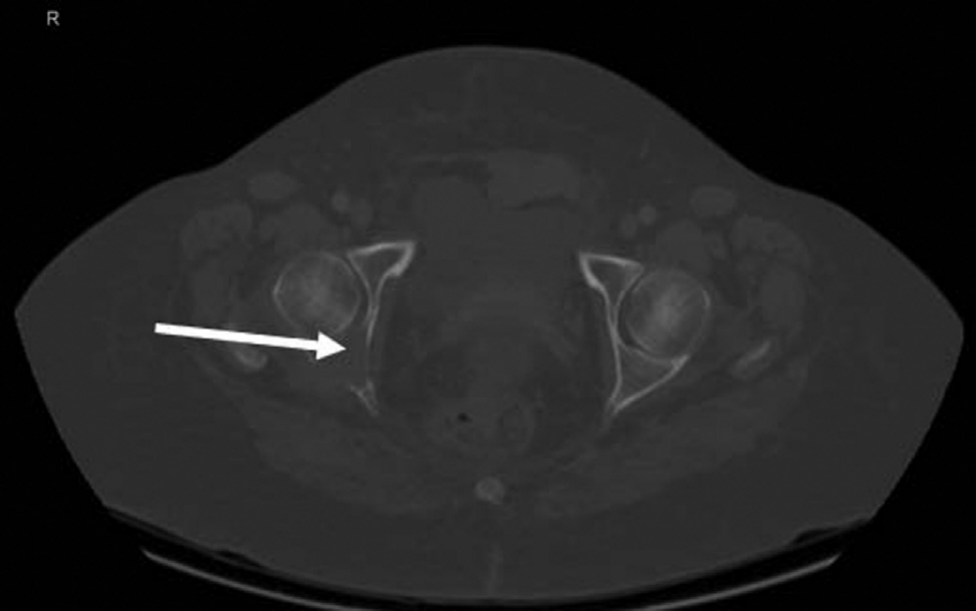
CT scan of the pelvis showing destructive lesion involving the right ischial tuberosity representing A metastatic deposits. CT, computed tomography.

**Fig. 2:**
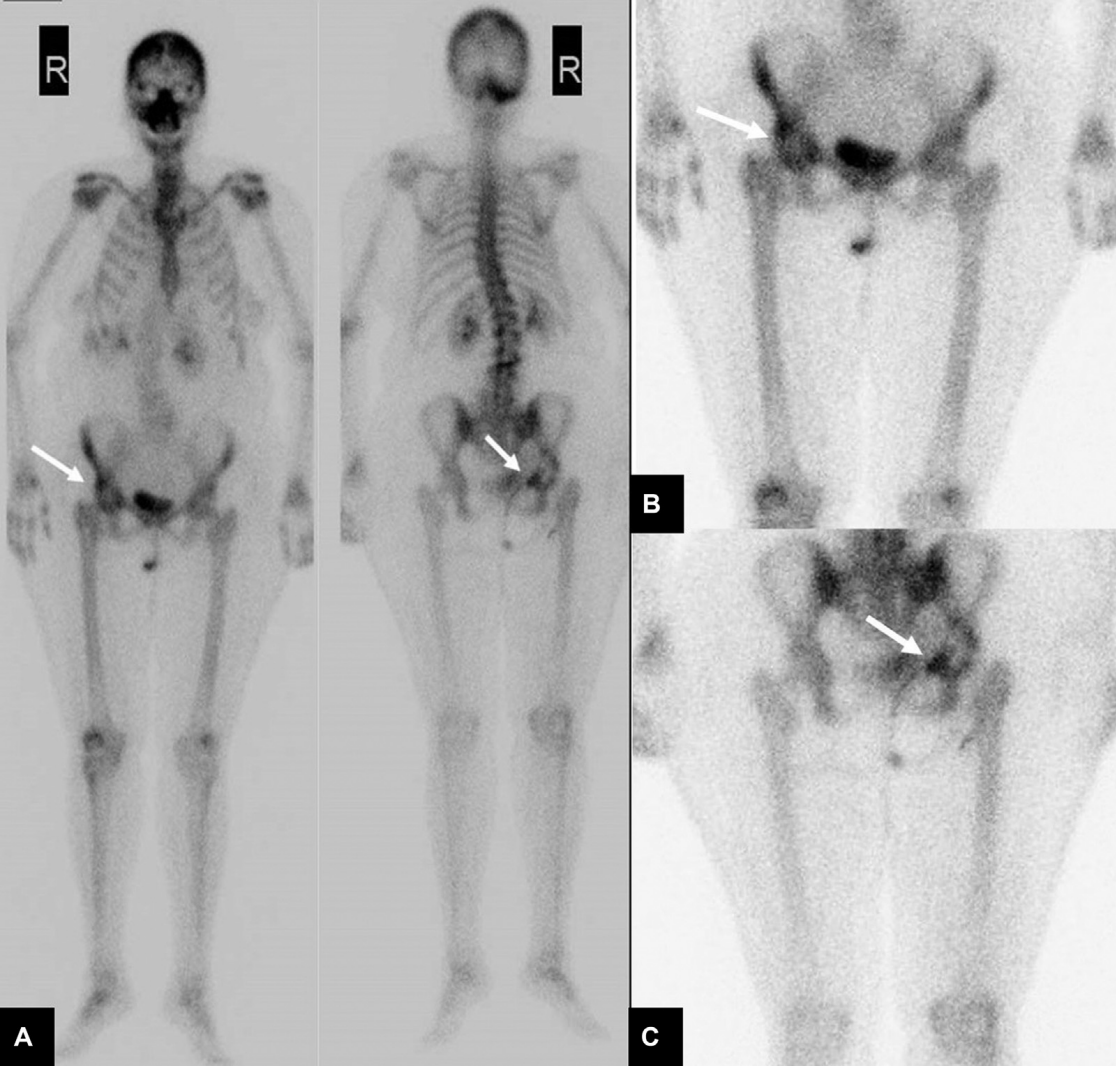
Whole body bone scan ( **A** ) anterior and posterior views. ( **B** ) Anterior spot view of pelvis. ( **C** ) Posterior spot view of pelvis. Images revealed active bone lesion within the right ischium highly suspicious for metastatic bone lesion.

**Fig. 3:**
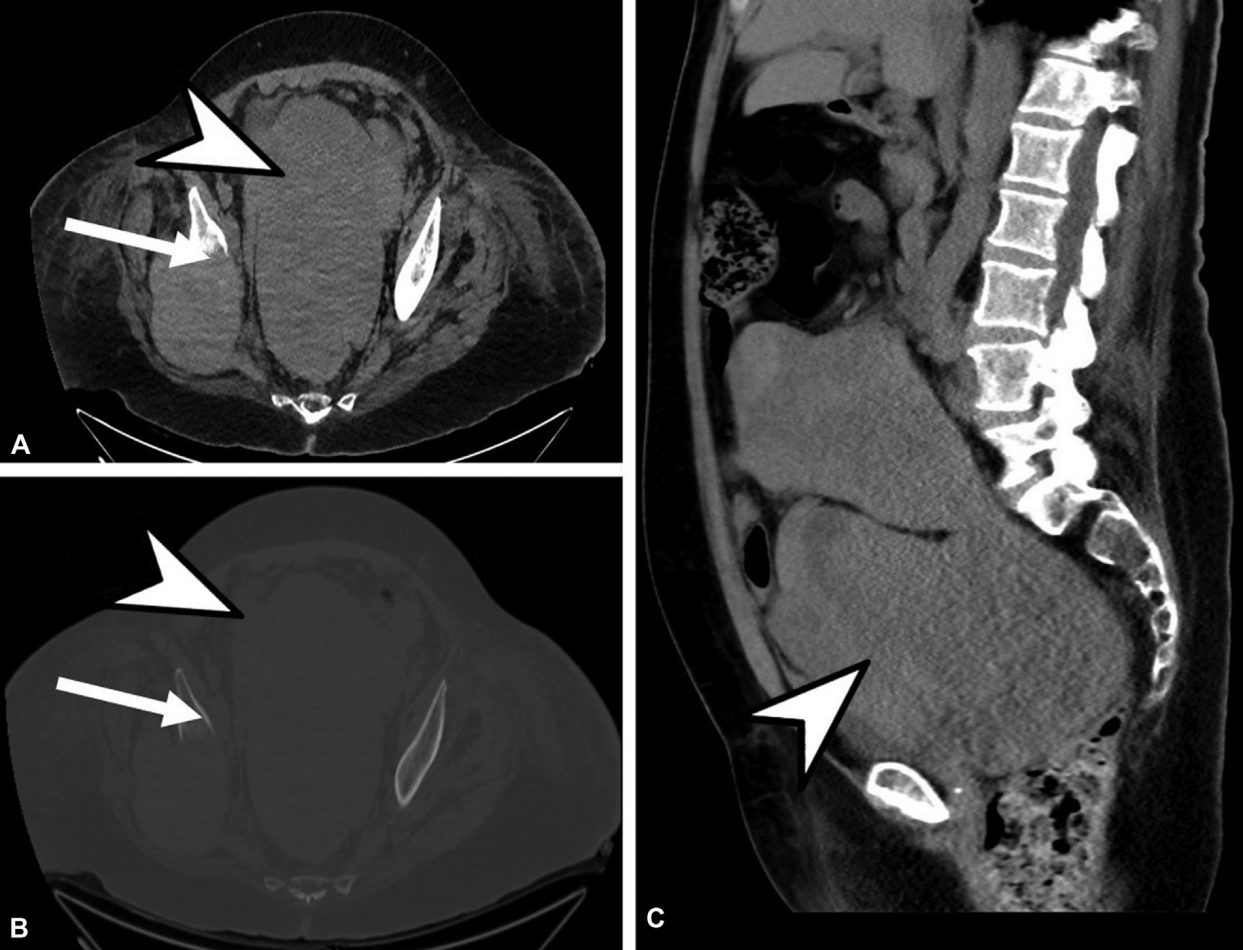
Abdominopelvic CT scan without contrast. ( **A** ) Axial view with soft tissue window, ( **B** ) sagittal view with soft tissue window, ( **C** ) axial view with bone window. All images demonstrate progression in size of the previously known pelvic mass lesion exerting pressure on urinary bladder and causing obstructive uropathy ( *arrow heads* ) along with more prominent osteolytic destructive right ischial bone lesion ( *arrows* ). CT, computed tomography.

In July 2021, the patient started complaining of pain on the right side of the pelvis along with symptoms of abdominal pain and distention that began to progress over time, affecting hip joint movement and causing severe anorexia for the patient. Whole-body computed tomography (CT) scan showed large multiloculated fluid collections within the pelvis with enhancing walls, raising the possibility of abscess. This was associated with evidence of bone and abdominal lymph node metastasis ( Fig. 1 ). In August 2021, the patient was referred to the nuclear medicine department to further assess the bone pain. A whole-body bone scan showed evidence of an active bone lesion within the right ischium, suspicious of a metastatic bone lesion ( Fig. 2 ). Chemoradiotherapy was planned but delayed due to rapid disease progression. Unfortunately, the patient developed obstructive uropathy, mandating admission to the intensive care unit. A follow-up CT scan at that time interval revealed that the previous multiloculated mass had grown in size, exerting pressure on adjacent structures and causing obstructive uropathy ( Fig. 3A, B ). The right ischial osteolytic lesion had also become more prominent since the previous scan ( Fig. 3C ). Subsequently, the patient developed urosepsis with multiple organ failure and succumbed 4 months after surgery.

## Discussion

The incidence of high-grade stromal sarcoma is extremely rare. The average age group of the presentation is between 40 and 55 years of age. 2 So, having this type of tumor at the age of 58 years with progressive involvement of the bone is a very rare clinical presentation of that tumor that has been reported only in a few cases before. HGESS symptoms are frequently atypical, leading to misinterpretation and misdiagnosis, as it can overlap and mimic uterine fibroid symptoms.

Vaginal bleeding, abdominal pain, abdominal distention, and pelvic mass are the most common clinical features associated with HGESS. Initially, the patient was misdiagnosed as having a uterine fibroid, but surgery confirmed the diagnosis with HGESS. Three months after surgery, the patient had right hip discomfort which was attributed to a prolapsed intervertebral disc rather than taken in the context of the underlying disease. This was actually due to bone metastasis from her malignant uterine tumor which was later confirmed by a whole-body bone scan.

The usual surgical intervention performed for such cases consists of total abdominal hysterectomy with bilateral salpingooophorectomy and systemic pelvic and para-aortic lymph node dissection which should not be performed in cases of unremarkable lymph node involvement. 4

At first, the patient was pathologically staged as T1Nx, but with distant metastasis discovered later on, the International Federation of Gynecology and Obstetrics (FIGO) staging for this tumor is IVB. 2 Because HGESS has a high risk of recurrence and metastasis, postoperative adjuvant therapy is mandatory.

Pelvic external radiotherapy has been widely used as an adjuvant therapy to help reduce the risk of pelvic recurrence after surgery. It has not been shown to improve overall survival which may be because of extra–pelvic metastases. 4

Pautier et al concluded that the API regimen (doxorubicin, ifosfamide, and cisplatin) of chemotherapy resulted in a statistical increase of 3-year disease-free survival rate in 81 patients with uterine sarcoma who received four cycles of chemotherapy of the API regimen followed by radiotherapy as compared with a control group of 18 patients managed only with postsurgical radiotherapy. 5 Gemcitabine combined with docetaxel or doxorubicin/ifosfamide, doxorubicin/dacarbazine, gemcitabine/dacarbazine, and gemcitabine/vinorelbine are the most commonly used adjuvant chemotherapy for HGESS. 6

Targeted treatment for ESS is still controversial due to a lack of a large-scale clinical database. Pazopanib is a multitarget tyrosine kinase inhibitor used in systemic therapy of sarcoma. 6 On the other hand, apatinib is an oral vascular endothelial growth factor (VEGFR-2) intracellular inhibitor that inhibits neovascularization in tumor cells via highly selective competition for the VEGFR-2 ATP binding site in affected cells. It carries potential antitumor effects and has been shown to be a promising therapeutic option in the near future. 7

Metastatic bone lesions in HGESS cases are extremely rare and have been reported in very few cases till now. 8 9 Metastasis to bone can definitely increase the rates of mortality and morbidity and needs careful evaluation and follow-up.

Huang et al reported a rare case of ESS with bone metastasis that also involved the right pelvic bone, skull, and multiple vertebrae and was treated with chemoradiation after surgery followed by L3 laminectomy and subtotal tumor resection in the vertebrae. It was concluded that any musculoskeletal complaints should raise the suspicion of tumor involvement to avoid misinterpretation and achieve the best outcome. 9

Date et al reported a case of metastatic ESS to the skull 1 year after tumor diagnosis and concluded that close follow-up and observation for patients with metastatic ESS should be performed periodically to assess this aggressive tumor and achieve the best outcome possible. 8

## Conclusion

Patients with ESS are prone to misdiagnosis due to tumor rarity and a lack of typical and specific clinical features. Even though bone metastasis is rare, it is important to note that any musculoskeletal complaints should raise the suspicion of tumor involvement to avoid misinterpretation and achieve the best possible outcome.
